# Effect of foot strike patterns and angles on the biomechanics of side-step cutting

**DOI:** 10.3389/fbioe.2024.1461247

**Published:** 2024-11-06

**Authors:** Fan Yi, Zhao Jianchao, Zhu Wen, Liu Ke, Lou Yantao

**Affiliations:** ^1^ School of Sports Health, Shenyang Sport University, Shenyang, Liaoning, China; ^2^ Department of Research and Medical, Shanghai Sports School, Shanghai, China; ^3^ Jining Health School, Jining, Shandong, China

**Keywords:** side-step cutting, foot strike pattern, cutting angle, biomechanics, anterior cruciate ligament

## Abstract

**Objectives:**

The study aimed to determine how foot strike patterns and cutting angles affect lower extremity (LE) kinematics, kinetics, and muscle activity during side-step cutting.

**Methods:**

Twenty male college sport athletes participated in this research. Three-dimensional motion analysis featuring ground reaction force (GRF) and electromyography (EMG) of the dominant leg was used. LE kinematics, kinetics, and EMG data parameters were obtained during a 45° and 90° side-step cutting involving rearfoot strikes (RFS) and forefoot strikes (FFS).

**Results:**

The significant foot strike pattern × angle interactions were observed for the ankle eversion range of motion (ROM) at the loading phase. Cutting of 90° had greater knee flexion ROM, knee valgus ROM, and knee varus moment compared to that of 45°. RFS cutting had greater knee flexion, hip flexion, knee valgus, knee varus moment, knee varus moment, and ankle eversion ROM. FFS cutting produced a lower vertical GRF, lateral GRF, and a loading rate. Both vastus medialis and vastus lateralis muscle activities were remarkably greater during cutting of 90° than 45°. At the loading phase, semitendinosus, biceps femoris, and the lateral head of gastrocnemius muscle activities during FFS cutting were considerably greater than those during RFS cutting.

**Conclusion:**

The FFS pattern can better protect the anterior cruciate ligament (ACL) and improve the flexibility of athletes by increasing the plantarflexion torque of the ankle. The injury risk also increases with the larger cutting angle. The EMG activities of semitendinosus and biceps femoris are vital for the stability of knee joint during side-step cutting, which helps reduce ACL stress during buffering.

## 1 Introduction

Anterior cruciate ligament (ACL) injuries are one of the most common injuries for athletes. More than 200,000 ACL injuries occur in America annually, seriously damaging the health of athletes (Weinhandl et al., 2014). A study by Walden, et al. on European professional soccer players found a consistent 6% annual increase in the ACL injury rate (Waldén et al., 2016). Athletes undergoing surgery are likely to miss a sports season or sports scholarships and have a higher risk of osteoarthritis and other pathological conditions (Freedman et al., 1998; Sepúlveda et al., 2017). After an ACL injury, more than 50% of athletes cannot return to previous sport performance (Donnelly et al., 2012).

Statistics show that side-step cutting occurs more than 100 times in a soccer game (Havens and Sigward, 2015a). Approximately 70% of ACL injuries result from non-contact situations and are correlated with decelerating and evasive cutting maneuvers (Boden et al., 2000; McNair et al., 1990). Different foot strike patterns and angles of the side-step cutting will impact the biomechanical parameters of LE. Study indicates that runners generate less ground reaction force (GRF) using forefoot strikes (FFS) than rearfoot strikes (RFS) (Lieberman et al., 2010). The RFS pattern will reduce the angle of joint flexion, produce a greater ground reaction force, weaken the cushioning function of lower limb joints, and lead to increased stiffness of lower extremity (LE). It mainly relies on the knee joint to absorb the external load, while the FFS pattern mainly depends on the ankle joint (David et al., 2017; Donnelly et al., 2017; Fox, 2018). Another predisposing factor for non-contact ACL injury is cutting angles. Cortes et al. discovered a greater knee valgus angle and lower knee flexion angle in 180° cutting compared to 45°, suggesting that it is more likely to put the knee in a valgus, extension position with a larger angle cutting, which increases tibialis anterior shear forces and thus increases ACL stress (Cortes et al., 2011). In terms of kinetics, the vertical, posterior, and lateral GRF increase by 21%, 87%, and 228%, respectively, in the 110° cutting compared to 45° (Jamison et al., 2012).

There is limited research on foot strike patterns and angles in cutting maneuvers, and most studies focus on analyzing a single factor. The interactions between these constructs have not been studied. Existing research only indicates that larger cutting angles and the biomechanical characteristics in the RFS pattern increase the risk of ACL injury. There is insufficient evidence to support that the change in foot strike patterns with an increased cutting angle will reduce the LE injury risk. Therefore, further research is necessary.

This study aims to evaluate how foot strike patterns and angles influence LE kinematics, kinetics, and EMG parameters during side-step cutting in order to provide theoretical support and practical assistance in the prevention of LE injuries during side-step cutting.

## 2 Methods

### 2.1 Research participants

Twenty male injury-free athletes (mean age, 23.1 ± 0.6 years; mean height, 175.1 ± 5.4 cm; mean weight, 68.5 ± 4.8 kg) participated in the study. Minimum power of the study required is 80% (α = 0.05) (Havens and Sigward, 2015b). *A priori* power analysis using G*Power 3.1.9.7 (Universitat Kiel, Germany) revealed that obtaining a power of 0.80 at an effect size of 0.30 for the medium effect of ANOVA and with an alpha level of 0.05 required a sample size of at least 17 subjects (Havens and Sigward, 2015a; Dai et al., 2015). Each attendee offered written consent. Shenyang Sport University Research Institutional Review Board approved this research.

### 2.2 Experimental setup

Three-dimensional kinematic data were recorded at 200 Hz using four high-speed cameras (Has-200R; Ditect, Tokyo, Japan). An EIMG 3D calibration frame (24 Marker points, EIMG, China) was used for static calibration before and after the experiment to avoid shifting and shaking. The landmarks were placed on the left and right sides of the ankle, knee, hip, shoulder, elbow, and hand. An embedded 2093 mm × 469 mm × 18-mm force plate (RSscan International, Olen, Belgium) enabled a synchronous recording of GRF data at 1,000 Hz. EMG data were recorded by ME6000-T8 surface electromyography (Mega Electronics, Kuopio, Finland) with a sampling rate of 1,000 Hz. Bipolar surface electrodes (Ag–AgCl) were placed on the following seven muscles: the vastus medialis (VM), vastus lateralis (VL), medial head of the hamstrings (MH), lateral head of the hamstrings (LH), tibialis anterior (TA), lateral head of the gastrocnemius (LG), and medial head of the gastrocnemius (MG). Each electrode was placed over muscle belly parallel to the muscle’s line of action with a center-to-center distance of 2.5 cm (Wang et al., 2018).

The data on kinematics and kinetics were collected via an out-of-machine synchronization approach. The mouse controlled the kinetics and sEMG instrument and a LED light that could be captured by camera 1. Clicking the mouse could turn on the force plate, sEMG, and light at the same time (Yantao et al., 2016).

### 2.3 Protocol

**FIGURE 1:**
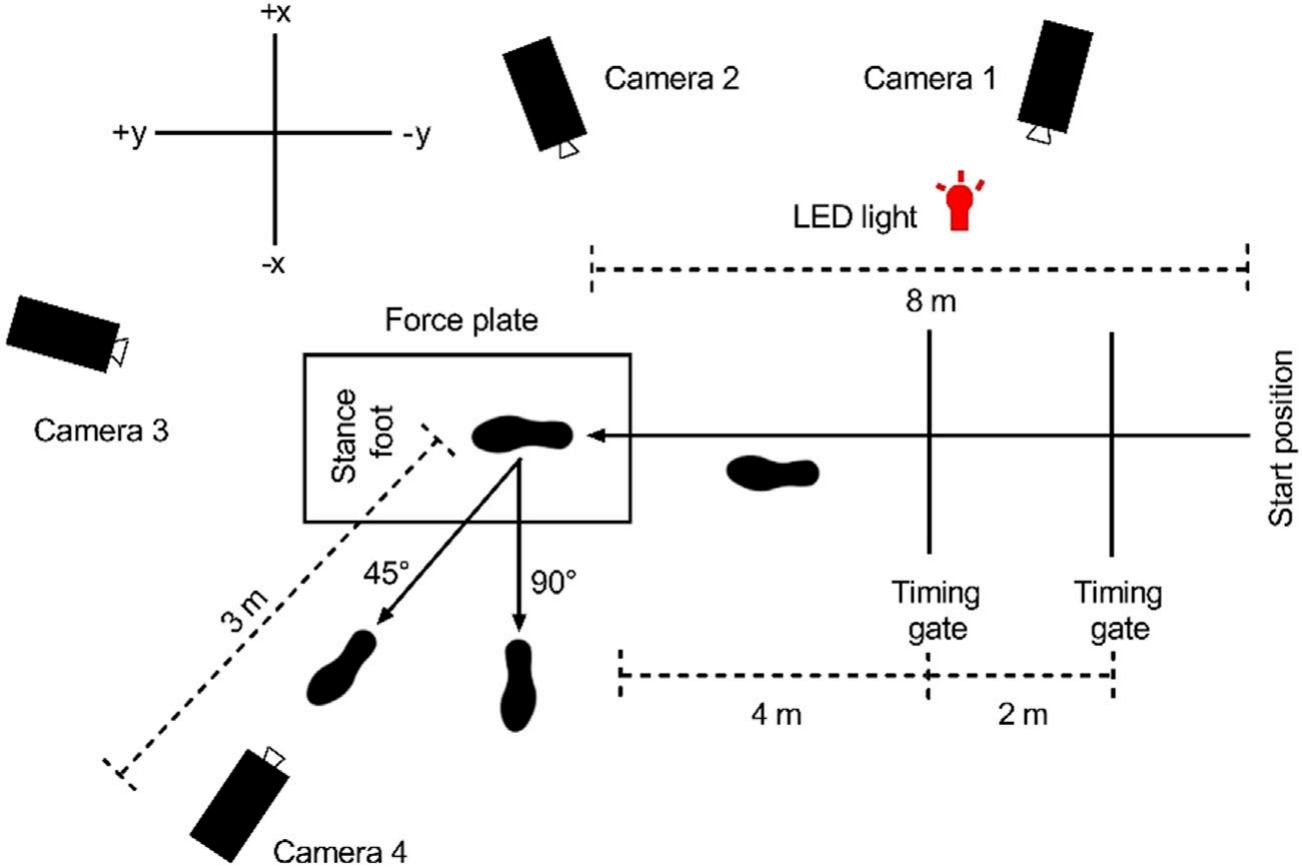
Experimental setup for side-stepping cutting trials.

Participants were provided spandex shorts and fitted with the same style of cross-training shoe (Nike Air Max 98, Portland, OR, USA). Prior to testing, all the participants performed a warming-up protocol for 5 min. One trial consisted of an 8-m runway toward the force plate, a side-step cutting on the force plate, and a 3-m sprint toward the endpoint (Figure 1). In agreement with another study, participants in this experiment also ran at 4 ms^−1^ ± 10% (Fain et al., 2019; Vanrenterghem et al., 2012). The approach velocity was monitored by two timing gates positioned 2 m apart. Four different conditions were examined, namely, making side-step cutting of 45° and 90° under the RFS pattern and FFS pattern. The FFS involved initial contact (IC) with toes followed by heels, and *vice versa*. During the side-step cutting, participants were instructed to plant their dominant foot on the force plate and then cut as quickly as possible toward the side opposite to the plant foot an angle of 45° or 90° in FFS and RFS patterns (Figure 1). A white tape placed at 45° and 90° from the original direction of progression was used to facilitate the cutting angle. The dominant leg was defined as the one that was used to kick a soccer ball (Peters, 1988). Each condition was completed three times. Foot strike patterns and angles were random during the experiment, and subjects were given a 60-s rest period between each action to prevent fatigue. At the end of the experiment, according to the action screening principle, an expert used a video playback to determine whether the changing angle and foot strike patterns met the standards and finally to determine whether the action was effective. A successful trial was defined as follows: (1) The running speed was within the range; (2) the foot strike pattern was accurate, and the dominant foot was completely on the force plate; (3) no EMG signal lost during the whole movement.

### 2.4 Data analysis

**FIGURE 2:**
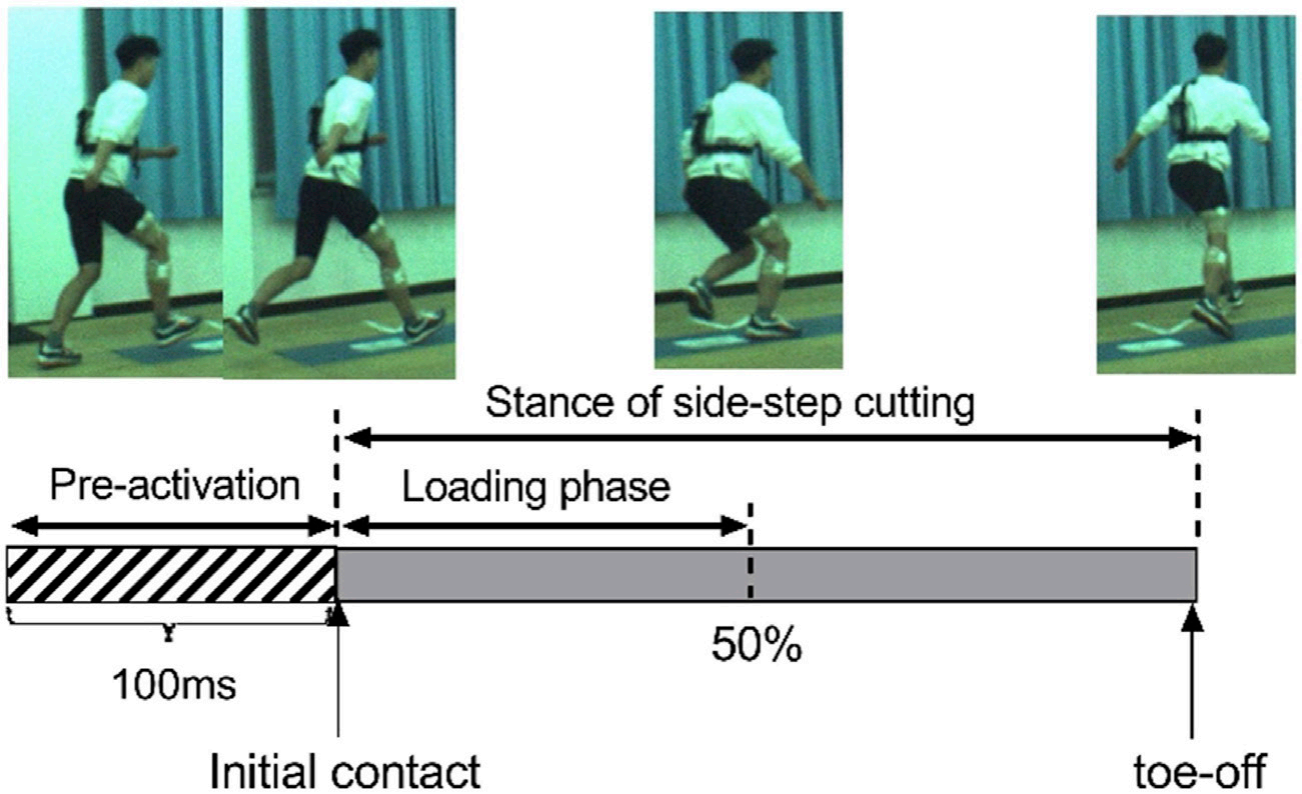
Stage division of side-step cutting.

All the video files were imported into Simi Motion 8.5.6 (Simi Reality Motion Systems GmbH, Unterschleissheim, Germany) and digitized by an experienced operator manually. Both sides of the body were digitized. To ensure data synchronization with other devices, digitizing started as the light is on and ended with toe-off. Adjustments were made necessary using the points-over-frame method, where each point was tracked through the entire sequence (Bahamonde and Stevens, 2006). The 3D direct linear transformation algorithm was used to reconstruct the 3D coordinates from each camera’s x- and y-image coordinates (Abdel-Aziz et al., 2015). Low-pass digital filtering was used to smooth the original data, and the fourth-order Butterworth low-pass filtering of 10 Hz was used to filter the original data. The variables included ankle plantarflexion, knee flexion, hip flexion, knee valgus, and ankle eversion angles at the pre-activity phase, IC, and loading phase (Figure 2).

Kinetics variables included vertical GRF (vGRF), posterior GRF (pGRF), lateral GRF (lGRF), loading rate knee extension moment, knee varus moment, and knee internal rotation moment. The IC of side-step cutting was identified as the first frame at which the vGRF exceeded 10N. The “loading phase” was identified as the moment from IC to the maximum knee flexion angle.

**FIGURE 3:**
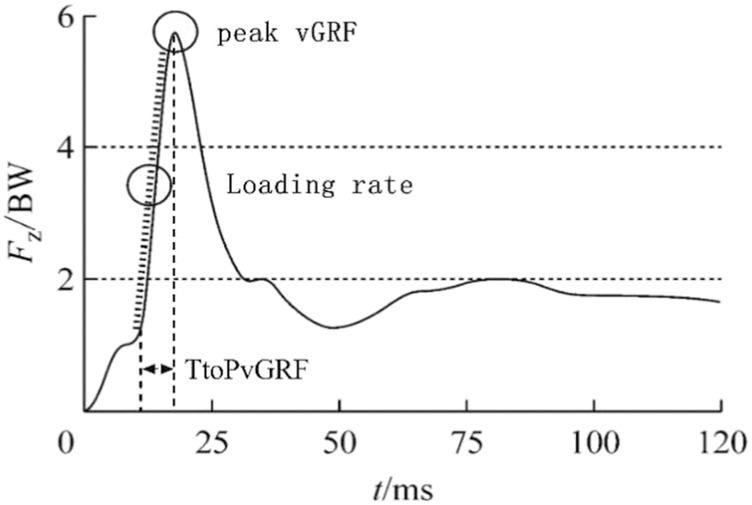
Schematic diagram of the loading rate.

Data were processed by a fourth-order low pass Butterworth filter with a cut off frequency of 10 Hz. The loading rate is defined as the slope of the line between the point where the force plate reaches 10N and the peak vGRF at the moment of foot contact (Figure 3). The calculation method is the peak vGRF divided by the time it takes to reach the peak vGRF (Gelalis et al., 2012). Kinetic measurement was normalized to body weight, allowing a comparison between subjects (Condello et al., 2016). All the kinematic and kinetic time-series waveform data were time-normalized to 100% of the stance phase and ensemble-averaged.

$$Loading\;rate\left(\%\right)=\frac{\text{PvGRF}}{\text{TtoPvGRF}}\times 100\%$$

The raw EMG data were imported into Megwin 2.4 software (Mega Electronics, Kuopio, Finland). All the EMG data were low-pass filtered at 250 Hz, high-pass filtered at 10 Hz, full-wave rectified, and smoothed by a zero-lag fourth-order Butterworth low-pass filter with a cut-off frequency of 30 Hz. The EMG signals were converted to root mean square (RMS) values, and they were recorded during 100 ms before IC and the first 100 ms of the cutting phase. The intervals were examined for the following reasons: (1) 100 ms before IC was appropriate for an individual pre-planned muscle recruitment strategy (Padua et al., 2006); (2) 100 ms after IC was the moment that ACL injuries occur frequently (Boden et al., 2000; Bencke et al., 2013; Koga et al., 2010). The activation order of the seven muscles at the pre-activity phase was found according to the activation points defined as the timepoint at which the EMG activity first exceeded two root mean squares of the average baseline activity in each muscle. It was recorded during the quiet period before the side-step cutting (Bai et al., 2019). The processed EMG was normalized to the peak activation of the respective muscles examined during the side-step cutting (% peak EMG during cutting) (Herbaut and Delannoy, 2020; Mizuno et al., 2009).

$$RMS\left[EMG\left(t\right)\right]=\sqrt{\frac{1}{T}\int_{t}^{t+T}{\text{EMG}}^{2}\left(t\right)\cdot \text{dt}}$$

### 2.5 Statistics

Statistical analysis was performed using SPSS 21.0 (IBM, Armonk, NY, USA) and Origin 9.0 (Origin Lab, Northampton, Massachusetts, USA), and graphs were performed by GraphPad Prism 7.0 (GraphPad Software, La Jolla, California). A two-way repeated measures ANOVA was used to test the main effects and interactions between foot strike patterns (RFS, FFS) and cutting angles (45°, 90°). Significant interactions were submitted to simple main effects, and the Bonferroni procedure was used for pairwise comparisons. If significant interactions did not exist, the main effects of the foot strike patterns and angles were analyzed. The effect size (*η* ^*2*^: partial eta square) was calculated for significant main and interaction effects. According to the evaluation standard of Cohen et al., when partial η^2^ is >0.4, >0.25, and >0.1, the effect is high, medium, and low, respectively (Havens and Sigward, 2015a). Effects were considered to be significant at *p* < 0.05.

## 3 Results

### 3.1 Kinematics

**FIGURE 4:**
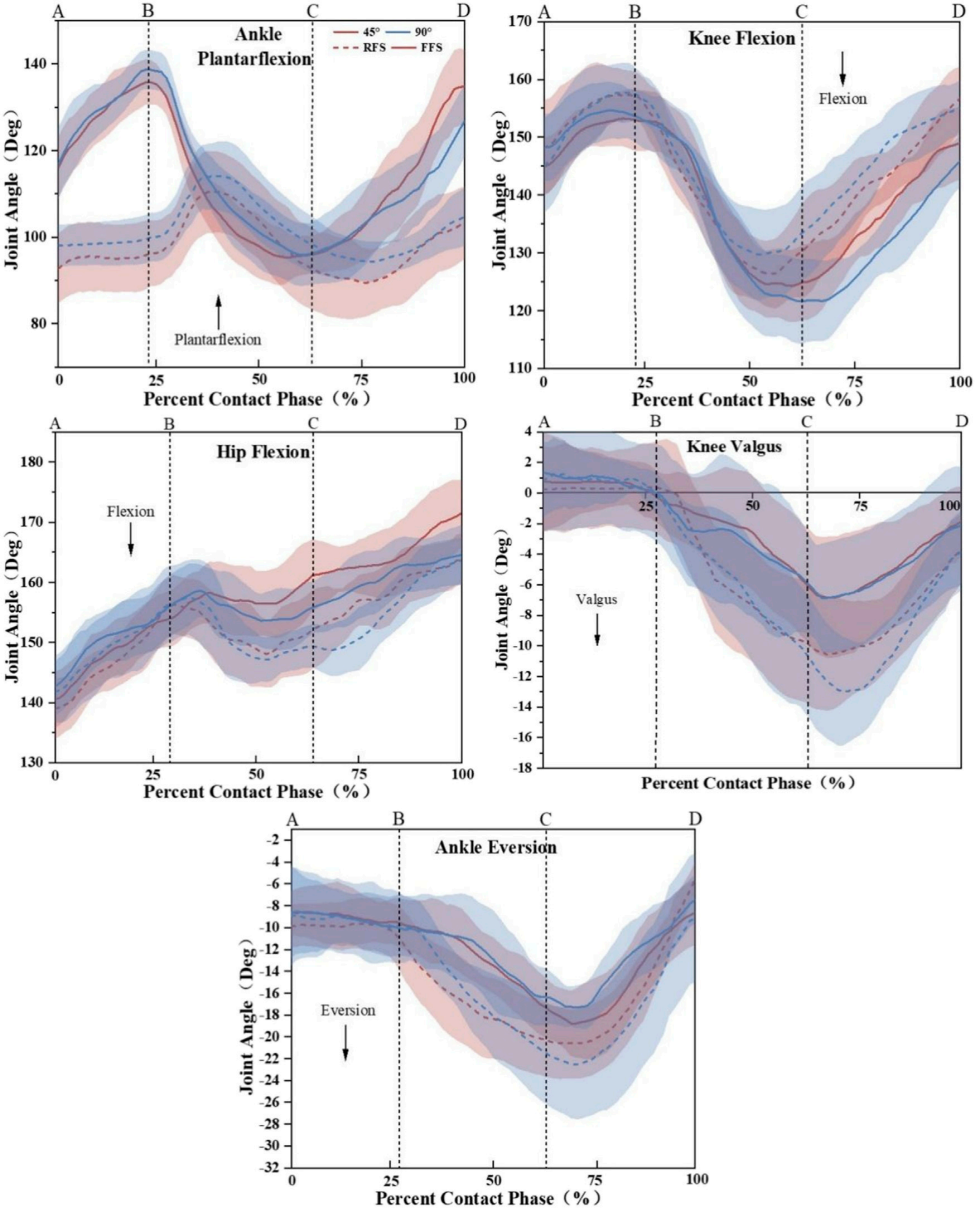
Mean and SD of the sagittal plane and frontal plane angular displacement at the ankle, knee, and hip joint for the FFS (solid) and RFS (dashed) patterns and the 45° (red) and 90° (blue) side-step cutting trials. **(A)** 100 ms before IC, **(B)** moment of IC, **(C)** initial 50% of the stance phase during side-step cutting, and **(D)** toe-off; (AB) pre-activity phase; (BC) loading phase.

As Figure 4; Table 1 show, the significant foot strike pattern ×angle interactions were observed for ankle eversion ROM (*p <* 0.01, ES = 0.57) at the loading phase. Specifically, RFS cutting had a greater ankle eversion ROM than FFS cutting at the angle of 90° (*p <* 0.01). When collapsed across the angle, RFS cutting produced a greater hip flexion (*p* < 0.05, ES = 0.20), knee valgus (*p* < 0.01, ES = 0.42), and ankle eversion ROM (*p* < 0.01, ES = 0.47) at the loading phase and lower knee flexion angle and ROM (*p* < 0.05, ES = 0.31, *p* < 0.05, ES = 0.63 and *p* < 0.05, ES = 0.25) at 100 ms before IC, IC, and the loading phase. When collapsed across the foot strike pattern, 90° cutting had greater knee flexion (*p* < 0.01, ES = 0.27) and valgus ROM (*p* < 0.01, ES = 0.15).

**TABLE 1:** Descriptive statistics (mean, standard deviation) for kinematics during 100 ms before IC, IC moment, and the loading phase between 45° and 90° by the foot strike pattern (Deg).

	FFS		RFS		*p*-value of two-way ANOVA		
	45°	90°	45°	90°	Foot strike pattern	Angle	Foot strike pattern × angle
100 ms before IC							
Ankle Df/Pf ROM^a^	20.0 (3.8)	21.9 (3.6)	−6.0 (1.7)	−6.5 (1.9)	0.00	n.s	n.s
Knee Flx/Ext ROM^a^	9.2 (1.2)	10.5 (2.3)	7.8 (1.9)	7.0 (0.8)	0.04	n.s	n.s
Hip Flx/Ext ROM	12.0 (1.6)	11.3 (1.9)	12.3 (2.7)	11.1 (1.3)	n.s	n.s	n.s
Knee Var/Val ROM	0.4 (0.0)	0.5 (0.0)	0.4 (0.0)	0.4 (0.0)	n.s	n.s	n.s
Ankle Inv/Eve ROM	0.3 (0.0)	0.4 (0.0)	0.4 (0.0)	0.4 (0.0)	n.s	n.s	n.s
IC							
Ankle Df/Pf angle^a^	135.7 (5.1)	138.6 (4.5)	96.3 (7.6)	99.9 (5.6)	0.00	n.s	n.s
Knee Flx/Ext angle^a^	31.2 (5.3)	30.4 (4.7)	22.1 (4.0)	22.3 (4.3)	0.02	n.s	n.s
Hip Flx/Ext angle	152.5 (4.2)	153.0 (4.8)	151.3 (5.3)	152.9 (6.2)	n.s	n.s	n.s
Knee Var/Val angle	0.6 (0.7)	0.4 (0.8)	0.9 (0.6)	0.4 (0.8)	n.s	n.s	n.s
Ankle Inv/Eve angle	9.9 (2.4)	9.8 (2.7)	9.5 (1.9)	9.8 (3.3)	n.s	n.s	n.s
Loading phase							
Ankle Df/Pf ROM^a^	−40.4 (3.3)	−42.6 (3.0)	17.4 (2.5)	15.5 (2.7)	0.00	n.s	n.s
Knee Flx/Ext ROM^a^,^b^	33.0 (2.7)	28.5 (2.5)	27.6 (3.9)	24.7 (2.9)	0.01	0.01	n.s
Hip Flx/Ext ROM^a^	8.2 (1.3)	8.0 (1.4)	10.2 (1.9)	9.9 (2.1)	0.02	n.s	n.s
Knee Var/Val ROM^a^ ^,^ ^b^	5.6 (1.9)	6.4 (1.0)	10.7 (1.1)	12.1 (1.4)	0.00	0.03	n.s
Ankle Inv/Eve ROM^a^ ^,^ ^c^	7.5 (1.0)	7.8 (1.1)	10.3 (1.4)	13.9 (1.3)	0.00	n.s	0.00

FFS, forefoot strikes; RFS, rearfoot strikes; IC, initial contact; Df/Pf, dorsiflexion (+)/plantarflexion (−); ROM, range of motion; Flx/Ext, flexion (+)/extension (−); Var/Val, varus (+)/valgus (−); Inv/Eve, inversion (+)/eversion (−).

^a^ Denotes a significant difference between the foot strike pattern.

^b^ Denotes a significant difference between the angle.

^c^ Denotes a significant interaction between the effect of the foot strike pattern and angle.

### 3.2 Kinetics

**FIGURE 5:**
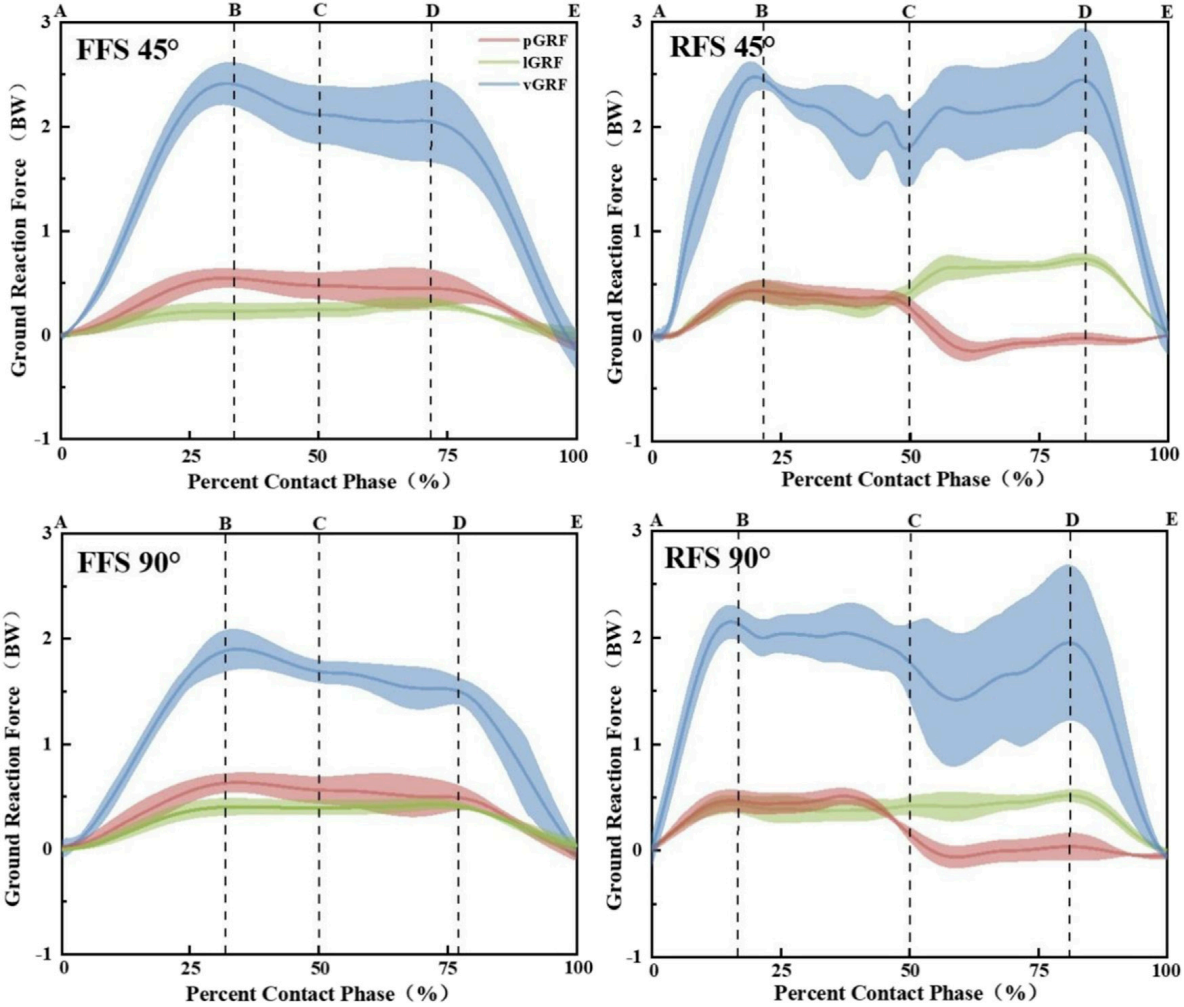
Mean and SD of the vGRF (blue), lGRF (green), and pGRF (red) for the FFS and RFS patterns and 45° and 90° side-step cutting trials. **(A)** Moment of IC, **(B)** moment of peak vGRF, **(C)** initial 50% of the stance phase during side-step cutting, **(D)** moment of the second peak vGRF, **(E)** moment of leaving the force plate; (AB) time to PvGRF; (AC) loading phase.

The GRF curves and kinetics parameters are presented in Figure 5; Table 2. A significant main effect of foot strike patterns and angles was found for the vGRF (*p* < 0.01, ES = 0.51 and *p* < 0.05, ES = 0.19), lGRF (*p* < 0.01, ES = 0.98 and *p* < 0.01, ES = 0.63), and loading rate (*p* < 0.01, ES = 0.63 and *p* < 0.01, ES = 0.56). A significant main effect of foot strike patterns was found for the pGRF (*p* < 0.01, ES = 0.33). When collapsed across the foot strike pattern, the cutting of 90° had greater lGRF compared to 45°; the cutting of 90° showed a lower vGRF and loading rate than 45°. When collapsed across the angle, RFS cutting produced a greater vGRF, lGRF, pGRF and loading rate than FFS cutting.

**TABLE 2:** Descriptive statistics (mean, standard deviation) for GRF and the loading rate between 45° and 90° by the foot strike pattern (N/kg).

	FFS		RFS		*p*-value of two-way ANOVA		
	45°	90°	45°	90°	Foot strike pattern	Angle	Foot strike pattern × angle
vGRF^a^ ^,^ ^b^	19.8 (3.3)	18.6 (3.9)	24.3 (1.8)	20.9 (3.0)	0.00	0.02	n.s
lGRF^a^ ^,^ ^b^	2.1 (0.3)	3.6 (0.4)	3.6 (0.4)	4.4 (0.4)	0.00	0.00	n.s
pGRF^a^	7.3 (0.5)	7.6 (0.5)	9.3 (0.6)	9.4 (0.6)	0.00	n.s	n.s
Loading rate^a^ ^,^ ^b^	23.8 (4.2)	20.1 (4.1)	31.7 (5.4)	25.1 (5.1)	0.00	0.00	n.s

FFS, forefoot strikes; RFS, rearfoot strikes; vGRF, vertical ground reaction force; lGRF, lateral ground reaction force; pGRF, posterior ground reaction force.

^a^ Denotes a significant difference between the foot strike pattern.

^b^ Denotes a significant difference between the angle.

^c^ Denotes a significant interaction between the effect of the foot strike pattern and angle.

The knee joint kinetics at the peak posterior ground are presented in Table 3. A significant main effect of foot strike patterns and angles was found for the knee extension moment (*p* < 0.01, ES = 0.30 and *p* < 0.05, ES = 0.19) and the knee varus moment (*p* < 0.05, ES = 0.13 and *p* < 0.05, ES = 0.21). When collapsed across the foot strike pattern, the cutting of 90° had a greater knee varus moment compared to 45°; the cutting of 90° showed a lower knee extension moment than 45°. When collapsed across the angle, the RFS cutting produced a greater knee extension moment and knee varus moment than FFS cutting.

**TABLE 3:** Descriptive statistics (mean, standard deviation) for knee joint kinetics at the peak posterior ground reaction force between 45° and 90° by the foot strike pattern (Nm/kg).

	FFS		RFS		*p*-value of two-way ANOVA		
	45°	90°	45°	90°	Foot strike pattern	Angle	Foot strike pattern × angle
Knee extension moment^a^ ^b^	1.96 (0.12)	1.1 (0.09)	2.3 (0.21)	1.8 (0.32)	0.00	0.04	n.s
Knee varus moment^a^ ^b^	0.4 (0.13)	0.8 (0.17)	0.6 (0.19)	1.0 (0.04)	0.03	0.04	n.s
Knee internal rotation moment	0.04 (0.11)	0.12 (0.08)	0.03 (0.04)	0.14 (0.06)	n.s	n.s	n.s

FFS, forefoot strikes; RFS, rearfoot strikes; VM, vastus medialis; VL, vastus lateralis semitendinosus; MH, medial head of the hamstrings; LH, lateral head of the hamstrings; MG, medial head of the gastrocnemius; LG, lateral head of the gastrocnemius; TA, tibialis anterior.

^a^ Denotes a significant difference between the foot strike pattern.

^b^ Denotes a significant difference between the angle.

^c^ Denotes a significant interaction between the effect of the foot strike pattern and angle.

### 3.3 EMG parameters

**FIGURE 6:**
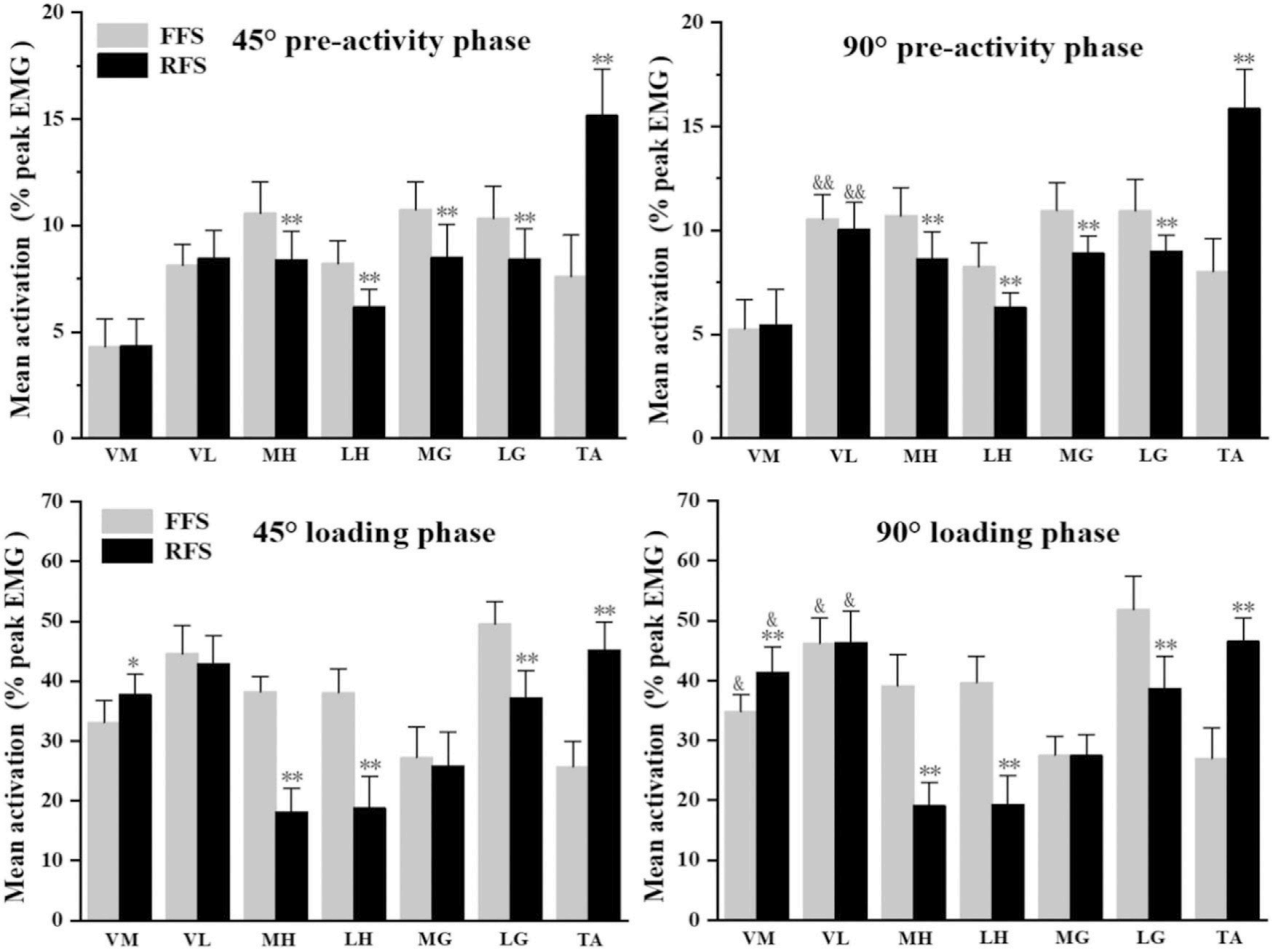
Mean muscle activation amplitude (% peak EMG during side-step cutting) and SD during the pre-activity phase and loading phase. * denotes a significant difference between FFS and RFS conditions. *p < 0.05 and **p < 0.01. & denotes a significant difference between 45° and 90° conditions. &*p* < 0.05, and *p* < 0.01.

The EMG parameters are presented in Figure 6; Table 4. At the pre-activity phase and loading phase, a significant main effect for some muscles appeared. When collapsed across the angle at the pre-activity phase, MH (*p* < 0.01, ES = 0.93), LH (*p* < 0.01, ES = 0.94), MG (*p* < 0.01, ES = 0.87), and LG (*p* < 0.01, ES = 0.82) muscle activities were significantly greater during FFS cutting than RFS cutting; the TA (*p* < 0.01, ES = 0.97) muscle activity was greater during RFS cutting. At the loading phase, MH (*p* < 0.01, ES = 0.98), LH (*p* < 0.01, ES = 0.77), and LG (*p* < 0.01, ES = 0.95) muscle activities were greater during FFS cutting than RFS cutting; TA (*p* < 0.01, ES = 0.97) and VM (*p* < 0.05, ES = 0.87) muscle activities were greater during RFS cutting. When collapsed across the foot strike pattern at the pre-activity phase, the cutting of 90° had greater VL (*p* < 0.01, ES = 0.91) muscle activity compared to 45°; at the loading phase, both VL (*p* < 0.05, ES = 0.48) and VM (*p* < 0.05, ES = 0.61) muscle activities were significantly greater during the cutting of 90° than 45°.

**TABLE 4:** Mean muscle activation amplitude (% peak EMG during side-step cutting) and SD during the pre-activity phase and stance phase between 45° and 90° by the foot strike pattern (%).

	FFS		RFS		*p*-value of two-way ANOVA		
	45°	90°	45°	90°	Foot strike pattern	Angle	Foot strike pattern × angle
Pre-activity phase							
VM	4.3 (1.1)	5.3 (0.6)	4.3 (1.5)	5.5 (0.8)	n.s	n.s	n.s
VL^b^	8.1 (0.8)	10.5 (1.5)	8.5 (0.7)	10.0 (0.7)	n.s	0.00	n.s
MH^a^	10.6 (0.7)	10.7 (0.7)	8.4 (0.9)	8.6 (0.8)	0.00	n.s	n.s
LH^a^	8.2 (0.7)	8.2 (0.8)	6.2 (0.5)	6.3 (0.4)	0.00	n.s	n.s
MG^a^	10.7 (0.7)	10.9 (0.6)	8.5 (0.9)	8.8 (0.7)	0.00	n.s	n.s
LG^a^	10.3 (0.7)	10.9 (0.8)	8.4 (0.9)	8.9 (1.0)	0.00	n.s	n.s
TA^a^	7.6 (1.0)	8.0 (0.8)	15.2 (1.3)	15.9 (1.1)	0.00	n.s	n.s
Loading phase							
VM^a^ ^,^ ^b^	33.1 (2.7)	34.7 (2.6)	37.7 (1.9)	41.3 (2.7)	0.01	0.02	n.s
VL^b^	44.5 (3.5)	46.1 (4.0)	42.8 (3.5)	46.3 (3.6)	n.s	0.04	n.s
MH^a^	38.1 (2.9)	39.0 (3.4)	18.1 (3.0)	19.1 (2.8)	0.00	n.s	n.s
LH^a^	38.0 (2.0)	39.6 (2.5)	18.7 (2.9)	19.3 (2.9)	0.00	n.s	n.s
MG	27.2 (2.6)	27.5 (2.3)	25.7 (3.2)	27.4 (2.3)	n.s	n.s	n.s
LG^a^	49.5 (2.1)	51.7 (3.0)	37.1 (3.2)	38.6 (3.7)	0.00	n.s	n.s
TA^a^	25.7 (2.9)	27.0 (3.4)	45.1 (5.3)	46.5 (2.7)	0.00	n.s	n.s

FFS, forefoot strikes; RFS, rearfoot strikes; VM, vastus medialis; VL, vastus lateralis semitendinosus; MH, medial head of the hamstrings; LH, lateral head of the hamstrings; MG, medial head of the gastrocnemius; LG, lateral head of the gastrocnemius; TA, tibialis anterior.

^a^ Denotes a significant difference between the foot strike pattern.

^b^ Denotes a significant difference between the angle.

^c^ Denotes a significant interaction between the effect of the foot strike pattern and angle.

**FIGURE 7:**
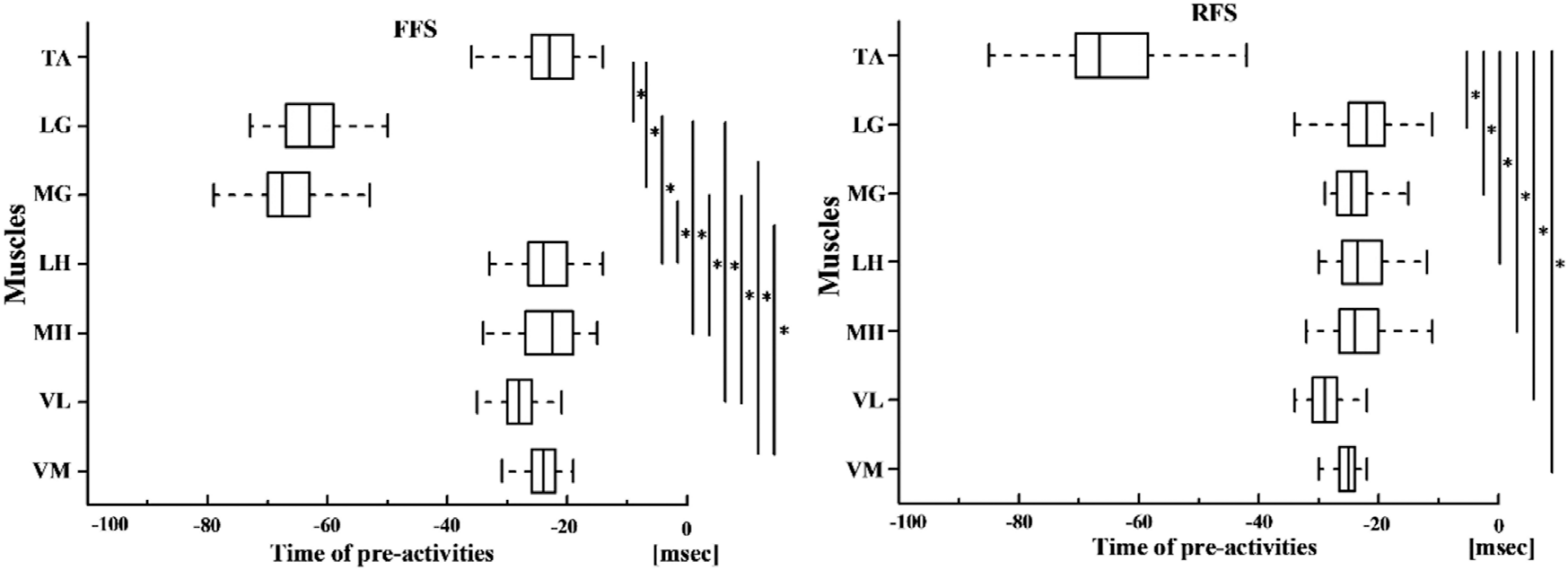
Pre-activities during FFS cutting and RFS cutting. Significant differences are indicated in bold lines.

In FFS cutting, the pre-activity timing of LG and MG was notably earlier compared with those of the muscles (TA, MH, LH, VL, and VM) (*p* < 0.01). In RFS cutting, the counterpart of TA was also significantly earlier compared with those of the muscles (LG, MG, LH, MH, VL, and VM) (*p* < 0.01) (Figure 7).

## 4 Discussion

This is the first study that investigates kinematics, kinetics, and EMG parameters during side-step cutting at different foot strike patterns and angles. LE biomechanics is essential for injury prevention for the reason that the combination of deceleration and change in direction is linked with side-step cutting that plays an important role in ACL injuries.

In agreement with Donnelly et al., the participants who used the FFS pattern experienced rapid plantarflexion of the ankle during the 100 ms before IC, while those using the RFS pattern experienced dorsiflexion of the ankle (Donnelly et al., 2017). It was observed that MG and LG activities are greater in the FFS pattern, and TA activity was greater in the RFS pattern at the pre-activity phase. Even at the loading phase, LG activity was still at a high level of activation, which might be related to the generation of ankle plantarflexion moments during cutting maneuvers of the final stage. Mizuno et al. and Sherbondy et al. reported that the pre-activation of the ankle plantarflexion in the FFS pattern before IC could help amortization, and the activation of the gastrocnemius could effectively reduce the forward movement of the tibia, which helped the stability of the knee joint (Mizuno et al., 2009; Sherbondy et al., 2003). Therefore, the FFS pattern can reduce the risk of ACL injury by increasing muscle activities of the gastrocnemius. A lower knee flexion angle and ROM at the pre-activity phase, the moment of IC, and the loading phase indicate that in the RFS pattern, the subjects’ knee is in a more extended position while performing the cutting tasks. Reduced knee flexion at IC has been proposed as a high risk for ACL injury (Hewett et al., 2005; Wojtys et al., 2002). With low knee angles (0°–30°), quadriceps can place enough strain on ACL to rupture it. In this study, it was discovered that the participants presented reduced knee flexion with the RFS pattern (22°) combined with the greater muscle activity of VM and VL at the loading phase. Additionally, the hip flexion ROM is greater in the RFS pattern; similar trends were reported in the previous study on gender comparisons (McLean et al., 2004). However, the interpretation of this result is controversial. Studies have shown that greater hip ROM might cause a larger knee valgus angle and moment, which could limit the capacity of the medial muscles of LE to fully resist the load of knee valgus (McLean et al., 2004; Havens and Sigward, 2015c). By contrast, Kipp et al. have found that active hip flexion could enable knee muscles to absorb energy better and minimize knee moment on other motion planes (Kipp et al., 2011). However, there is no reduction in the knee valgus angle in the RFS pattern in our research. Therefore, more investigation is required including the joint moment and muscle strength.

This study found a smaller knee flexion angle at the moment of IC with 45° and 90° cutting under the RFS pattern. The study by Cortes et al. similarly showed that the knee flexion angle decreased with the use of the RFS pattern during cutting (Cortes et al., 2012). This may be because the reduction of the knee flexion angle will make the lower limb maintain a more upright posture, that is, the “hard landing” strategy is adopted. At this time, the activity of the quadriceps femoris is increased, the knee joint is displaced forward, and the anterior tibial shear force is increased, and the ability of the lower limb to reduce the external load is weakened (Blackburn and Padua, 2009). During side-step cutting, the coronal and horizontal planes of the knee joint will be in different motion states during the IC, which indicates that the lower limb will adjust accordingly, according to the landing pattern. The findings of this study are partially supported by the finding that the RFS pattern had a greater knee valgus and internal rotation angle when performing in 45° cutting and a smaller knee valgus angle when performing in 90° cutting. The knee valgus angle is a strong predictor to ACL injuries (Fox, 2018; Ogasawara et al., 2020). It is hypothesized that RFS cutting and a sharper angle would increase the knee valgus and ankle eversion ROM, which is partially proved by the results of this study. First, knee valgus and ankle eversion ROM tend to be smaller during FFS cutting, which is accordant with the results of Yoshida et al. (2015). The FFS pattern might help athletes better align their LE to reduce the moment arm of the GRF in the frontal plane (Kristianslund et al., 2014). Therefore, the FFS pattern is relatively safe. Second, knee valgus and flexion ROM are only greater in a sharper angle in this study. The kinematics of ankle is not affected by the cutting angle. This might be because the lower knee flexion ROM in 90° cutting results in a greater knee valgus ROM, and greater power and energy could be absorbed by the knee joint, thus reducing the load on the ankle joint. Based on the findings of strike pattern × angle interactions, it proved that RFS cutting has a greater ankle eversion ROM only at the angle of 90°. Differences between different foot strike patterns for each angle suggest that the landing techniques present differentiated characteristics and that the injury mechanism in a sharper cutting angle may depend on the combination of the foot strike pattern and cutting angle.

Relevant studies have shown that injury is prone to occur within 50 ms after IC, and the first peak posterior ground reaction force and the peak of knee extension moment may also occur at this stage (Krosshaug et al., 2007). Some studies have also shown that the 45° or 90° cutting with the RFS pattern has a larger knee extension moment and knee varus moment than the FFS landing, which are consistent with the results of this study (David et al., 2017; Donnelly et al., 2017). This is because the RFS pattern relies primarily on the knee joint for posture adjustment, and an increase in the knee extension moment leads to greater anterior tibial shear force, which places more load on the knee joint. A larger valgus/varus moment also increases the load on the ACL. Combined with the research points of Schreurs et al., this may be due to the fact that the FFS pattern can provide the athlete with a more vertical sagittal lower limb force line, thereby reducing the risk of injury caused by the knee valgus moment (Schreurs et al., 2017). Cortes et al. found that when the cutting angle increased, the knee varus moment also increased (Cortes et al., 2011). Our study also reached the same conclusion. It is possible that to reduce the load on the sagittal plane of the knee joint, athletes pre-rotate and lateral bend their trunk to make it more inclined to the cutting direction, and the rotation of the trunk drives the rotation of the lower limbs, thereby increasing the load on the coronal and horizontal planes of the knee joint (Havens and Sigward, 2015a; Sigward and Powers, 2007). Therefore, in order to prevent excessive load on the knee joint, we believe that for athletes with a history of knee injury, we should try to avoid RFS patterning in cutting.

Side-step cutting performed with a larger cutting angle is common in multi-directional sports and requires a greater impulse to accomplish (Schreurs et al., 2017; Bloomfield et al., 2007), which is reflected in a higher GRF and knee valgus angle. In agreement with the previous study, larger lGRF was also observed in 90° cutting (Sigward et al., 2015). In the case of a sharper angle, subjects had to brake to a greater extent while accelerating in a new direction, so the lateral component force of GRF increases with a sharper angle. Schreurs et al. (2017) revealed that lGRF and pGRF during cutting contribute to as much as 20% of the variance in knee frontal plane loading, which is related to an increased ACL injury risk. Contrastively, vGRF do decrease when cutting to a sharper angle. The result is somewhat surprising, given that previous research reported that cutting with a sharper angle has greater vGRF (Sigward et al., 2015; Schot et al., 1995). It might be explained by the fact that when cutting to sharper angles, athletes have to lean more toward the corner to meet the greater redirection demand. The vGRF becomes less perpendicular to the ground as the center of pressure is further away from the center of mass. Most studies have demonstrated that the ACL injury risk tend to be high during RFS cutting (Cortes et al., 2012; Koga et al., 2018). Compared to the RFS pattern, runners using the FFS pattern generate less GRF and knee loading (Kulmala et al., 2013). These findings support the results of our study. We found that RFS cutting produces a greater vGRF, lGRF, and pGRF. The RFS pattern was characterized by only one impact transient, and rapid changes in GRF may increase the injury risk of soft tissue around the knee (Donnelly et al., 2017). Increased pGRF and lGRF at IC have been theorized to increase the strain on ACL by enhancing the proximal anterior and lateral tibia shear force, which creates an anterior movement of the tibia, thus increasing the strain on the ACL (Sell et al., 2007). The difference between FFS and RFS patterns consists in the position of the GRF action point (center of pressure, COP) in the sagittal plane along the axis of tibial rotation. In the RFS pattern, the COP is located posterior to the tibial rotation axis, and the lGRF acting on the heel first generates a valgus moment around the sagittal axis of the knee while generating an internal rotation moment on the tibial rotation axis. The FFS pattern produces the opposite moment along the tibial rotation axis as the COP is located anterior to the tibial rotation axis. Therefore, the RFS pattern produced combined knee valgus and tibial internal rotation angles more frequently than FFS. The loading rate can better reflect the relationship between the impact and injury (Michel et al., 2010; van der Worp et al., 2016). The results of our study and existing studies show that the maximum loading rate in the FFS pattern is significantly lower than that in RFS, indicating that the FFS pattern is more safe (Lieberman et al., 2010). However, we did not find that the loading rate increased with a sharper angle, which might be related to body preorientation and cutting speed. However, it does not mean that the safety of larger angle side cutting is higher, considering the increased knee valgus angle, and sharper cutting angles may still place the knee at high risks.

Furthermore, some significant differences were found between foot strike patterns and angles in RMS data during side-step cutting. Specifically, hamstring activation considerably decreased in RFS cutting during the pre-activity phase and loading phase. At the loading phase, the contraction of the quadriceps and hamstrings increases the pressure on the knee joint and promotes its stability (MacWilliams et al., 1999). The hamstrings can reduce the stress on ACL by limiting the forward movement of the tibia (Imran and O'Connor, 1997). Therefore, the lack of hamstring strength may be one of the key factors of ACL injuries, which is also supported by Zebis et al. (2009). Athletes with low pre-activation of the biceps femoris during side-step cutting have an increased risk of non-contact ACL injuries. Therefore, the injury risk of the RFS pattern is high, and it is essential to increase muscle activities of hamstrings while activating quadriceps. Another finding was that in 90° cutting, VL muscle activities significantly increased at both two phases, while VM muscle activities only increased during the loading phase. The eccentric contraction of the quadriceps at braking phases helps absorb strong external forces, and the concentric contraction in pedaling and extension phases accelerates the body’s rotation, which causes the increase in VM and VL muscle activities at sharper angles. However, there was no increase in hamstring and gastrocnemius muscles activities, a key factor to cause injuries as well. The single contraction of quadriceps will inevitably lead to the forward movement of the tibia and increase the tension of ACL. Therefore, it is strongly recommended that athletes use the FFS pattern as much as possible in side-step cutting and try to strengthen the concentric contraction of hamstrings.

### 4.1 Limitations

Nevertheless, there are two limitations in this research. First, side-step cutting is completed under unanticipated conditions due to its sudden and explosive characteristics. As a result, high-frequent cutting maneuvers are a must to cope with unanticipated external stimuli in many antagonistic sports events (Brown et al., 2009; Kim et al., 2014). Thus, future research will focus on the biomechanical characteristics under unanticipated circumstances. Second, we captured and analyzed the subjects’ kinematics, kinetics, and muscle activities while running at 4.0 m/s, which was within the range of training speeds. Vanrenterghem et al. (2012) also found a progression speed of 4.0 m/s, the most suitable for investigating knee loading mechanisms associated with a dynamic side-step cutting. However, speed cannot be constant in the whole process. Therefore, results of this study might not reflect subjects’ biomechanical parameters at their preferred pace or another speed rather than at 4.0 m/s.

## 5 Conclusion

The FFS pattern can better protect the ACL and improve the flexibility of athletes by increasing the plantarflexion torque of the ankle. The injury risk also increases with a larger cutting angle. The EMG activities of MH and LH are vital for the stability of knee joint during side-step cutting, which helps reduce ACL stress during buffering. We suggest that we should focus on strengthening neuromuscular intervention training, such as muscle strength and activation exercises of the hamstring.

## Data Availability

The original contributions presented in the study are included in the article/supplementary material; further inquiries can be directed to the corresponding author.
